# Ethical complexities of screening for depression and intimate partner violence (IPV) in intervention studies

**DOI:** 10.1186/1471-2458-11-S5-S3

**Published:** 2011-11-25

**Authors:** Victoria J Palmer, Jane S Yelland, Angela J Taft

**Affiliations:** 1 Primary Care Research Unit, The Department of General Practice, The University of Melbourne, Carlton Victoria 3056, Australia; 2 Healthy Mothers Healthy Families research group, Murdoch Children’s Research Institute, Flemington Road, Parkville Victoria 3053, Australia; 3 Mother and Child Health Research, La Trobe University, 215 Franklin St, Melbourne, Victoria 3000, Australia

## Abstract

**Background:**

Intervention studies for depression and intimate partner violence (IPV) commonly incorporate screening to identify eligible participants. The challenge is that current ethical evaluation is largely informed by the four principle approach applying principles of beneficence, non-maleficence, and respect for justice and autonomy. We examine three intervention studies for IPV, postnatal depression (PND) and depression that used screening from the perspective of principlism, followed by the perspective of a narrative and relational approach. We suggest that a narrative and relational approach to ethics brings to light concerns that principlism can overlook.

**Discussion:**

The justification most commonly used to incorporate screening is that the potential benefits of identifying intervention efficacy balance the risk of individual harm. However, considerable risks do exist. The discovery of new information may result in further depression or worries, people might feel burdened, open to further risk, unsure of whether to disclose information to family members and disappointed if they are allocated to a control group. This raises questions about study design and whether the principle of equipoise remains an adequate justification in studies with vulnerable groups. In addition, autonomy is said to be respected because participants give informed consent to participate. However, the context of where recruitment is undertaken has been shown to influence how people make decisions.

**Summary:**

The four principles have been subjected to criticisms in recent years but they remain prominent in public health and medical research. We provide a set of simple, interrogative questions that are narrative and relationally driven which may assist to further evaluate the potential impacts of using screening to identify eligible research participants in intervention studies. A narrative and relational based approach requires seeing people as situated within their social and cultural contexts, and as existing within relationships that are likely to be affected by the results of screening information.

## Background

Participating in health care intervention studies can affect more than the future course of people’s health. Considerable attention has been given to ensuring that individuals provide informed consent with their autonomy and privacy protected in research studies. However, intervention studies, particularly those in depression and intimate partner violence (IPV), raise additional ethical considerations beyond autonomy, privacy and justice. The challenge is that current ethical evaluation of intervention studies in primary medical care is largely informed by Beauchamp and Childress’s four principle approach. This is articulated by the principles of beneficence, non-maleficence, respect for justice and autonomy; and is referred to as ‘principlism’ [1].

Under principlism moral deliberation is guided by: beneficence as ‘the obligation to act to provide benefits (positive) and to balance benefits, risks and costs to produce overall results (utility)’; non-maleficence as ‘the obligation to avoid causing harm’; respect for autonomy as ‘the obligation to respect the decision-making capacity of the autonomous person’; and justice as ‘the obligation of fairness in the distribution of benefits and risks’ [2]. The principles are flexible in their interpretation and embrace tenets of two foundational normative ethical theories: deontology (duty based ethics) and utilitarianism (produce the greatest good for the greatest number) [3].

The four principles have been subjected to criticisms in recent years but they remain prominent in public health and medical research in spite of recent developments in public health to explore relational ethics [4] and other ethical frameworks [5]. Whilst some advocate that principlism is an easy to apply template that makes organizational sense [6], our paper advocates for the need to move beyond only principlism in the assessment and ethical evaluation of complex public health interventions, such as the use of screening within intervention studies. We do this by examining how screening to identify eligible research participants was used in three intervention studies for IPV, postnatal depression (PND) and depression. We suggest that a narrative and relational based approach to this problem brings to light concerns that principlism can overlook.

## The ethics of incorporating screening in intervention studies

Screening is a practice where individuals are investigated to detect unrecognized disease or its precursors [7], and recently to identify probable symptoms of psychosocial conditions like depression or to detect abuse and violence. Routine and universal screening for psychosocial issues in the general population is a controversial practice [8-10], which makes its use within intervention studies questionable. In routine population-based screening, ‘sufficient risk for further investigation or direct prevention’ is identified [11], however in intervention studies, especially if they are randomized controlled trials (RCTS), participants may not receive prevention or treatment options. This makes the use of screening within psychosocial studies testing for intervention effectiveness a complex moral issue.

Possible reasons for psychosocial interventions are ‘diverse problems such as childhood abuse and neglect, postnatal depression (PND), social isolation, and partner abuse’ [12]. Many of these problems co-exist and are closely associated resulting in intervention studies overlapping. Available evidence indicates that child abuse is a risk factor for intimate partner violence (IPV), depression is one of the most common sequelae of IPV [13,14], and PND occurs at a rate of 13% in new mothers during the early months post delivery; some of which can be related to IPV [8,15].

The view that routine and universal screening is a simple, quick and cheap method to improve the quality of care for depression [16] may provide an attractive solution for governments to curtail projected increases in disease burden [17]. Emerging evidence suggests that screening for PND does not represent value for money predominantly due to additional costs of managing women incorrectly diagnosed as depressed (false positive scores) [18]. However, the United States Preventative Task Force (USPTF) review of studies for depression including screening indicated some improvements in recognition, treatment and health outcomes for patients [19]. Since the USPTF and others who have conducted systematic reviews of IPV screening have not found sufficient evidence to recommend universal screening, its use within intervention studies remains somewhat open to discussion [20,21]. The contradictions about screening effectiveness and inadequate evidence for follow-up interventions when there are highly correlated psychosocial problems raised by these reviews suggests ethical complexities exist about its use within public health intervention studies.

The evidence base regarding the impact of screening and its uses in intervention studies is still in nascent stages. Progress for measuring the impact of IPV screening for example has been said to be impeded by three assumptions: that completing a screening test has no impact on individuals (tools are neutral), assessing screening by retrospective rather than prospective analysis is suitable and that there is a lack of agreed appropriate intervention for IPV once identified [10]. These assumptions equally apply to measuring the impacts of screening for depression and PND in intervention studies. While there is limited information about the impact of screening in intervention studies, the technique is commonly used in depression, PND and IPV studies particularly as a method to identify or recruit eligible research participants.

The question of whether to incorporate screening within intervention studies has received very little attention. Studies of IPV [9,22] and postpartum depression [8] have indicated that stigma, fear of disclosure and beliefs about the limitations of management may lead women to deliberately under-report symptoms or factors. Screening for psychosocial problems also raises the question of surveillance, especially for women experiencing IPV or depression concerned about the removal of children by child protection services or partners accusing them in court of being unfit mothers [23]. Therefore, being screened -- even at the level of determining eligibility for a study and not as part of an intervention itself -- is complicated.

Further considerations about using screening for recruitment in an intervention relate to whether screening tools provide accurate and efficient identification of potential risk or cases because of the detection of false positives and false negatives [17]. There is uncertainty around identifying the most suitable tool, given that for IPV alone there are more than 33 tools available to measure abuse [10]. Studies also need to consider who will administer screening tools and interpret results [17]. Indeed, some question if ‘screening’ is the appropriate term to use in IPV research since the complex and hidden nature of IPV belies the pre-symptomatic disease states for which screening tools are generally designed [10]. In additional file 1 we outline three intervention studies where screening was used to recruit or identify eligible participants into randomized controlled trials (RCTs) for depression, PND and IPV [24-26].

Study one is a pragmatic randomized controlled trial (RCT) in antenatal clinics to deliver a Preparing for Parenthood structured intervention to reduce risk factors for PND [24]. Study two is a multimodal counseling intervention RCT to improve postpartum outcomes in African American mothers [26]. Study three is a RCT to improve rates of depression, hopelessness and suicide ideation in elderly patients by placing depression health specialists in primary care practices [25]. Study one and two comparison groups were assigned to treatment as usual (TAU) standard care processes [24,26]. Study three also assigned participants to TAU control groups however individuals were informed that they qualified for a diagnosis of depression if they screened positive and they were referred back to their primary care provider who was provided with a written assessment of the person’s psychiatric condition and any presence of suicidal ideation or hopelessness [27]. All three studies targeted vulnerable groups: women in their first pregnancy, poor pregnant African-American women and elderly patients. The fact that participants were screened and identified as at-risk of depression, PND or IPV raises some questions about the suitability of TAU for the comparison groups; an issue that has been given attention by a number of authors previously [27-30].

The three studies are drawn upon to demonstrate the ethical issues that emerge from using screening to identify or recruit for intervention studies; our analysis does not focus on the interventions that were subsequently delivered to participants. The issues are ethically evaluated first by using principlism and subsequently from the perspective of narrative and relational theories.

## Ethical issues raised by screening in the three study examples – the view of principlism

Each of the studies in additional file 1 used screening as a method to identify and recruit eligible participants. Reynolds et al. [25] randomly sampled 50% of patients aged 60-74 years and 100% aged ≥75 years from primary care settings and administered the Center for Epidemiological Studies of Depression (CES-D) scale by telephone. Those who screened positive (≥ 11 score) were invited to meet with a research assistant on their next visit to a practice to learn more about the study, and if willing, complete a formal assessment to determine eligibility. Brugha et al. [24] asked women in their first pregnancy to complete the ‘Pregnancy and You’ screening questionnaire at their antenatal appointments. If they screened positive (presence of any one of six depression items) women were invited to participate in the intervention. El-Mohandes et al. [26] screened women by audio computer-assisted survey interview (ACASI) to determine eligibility of women with reported risks in designated areas of depression, smoking, environmental tobacco smoke exposure and IPV. Depression screening was determined using the Beck Inventory II and IPV by asking two questions related to physical/sexual harm and being afraid of a partner.

Additional file 2 presents some of the key arguments that a principle based approach would cover about the issue of whether to screen or not screen to determine eligibility for, or to recruit to an intervention. In the next section we expand on these key arguments by reference to the three studies to explore the ethical issues.

### Beneficence – balancing benefits against risks

Beneficence obliges researchers to act to provide benefits to research participants (positive benefits) and for these benefits to be balanced against any risks and costs (utility). Benefit is often determined by the concept of substantial benefit which refers to ‘an outcome that now or in the future might be regarded by the [research participant] as worthwhile’ [31]. All three studies could provide outcomes that are worthwhile now or in the future. The justification for many intervention studies testing for effectiveness is based on the premise of utility – that there will be positive benefits to the wider population if an intervention is identified as being effective. For example, earlier detection of suicide ideation may prevent serious individual harm for the elderly, the identification of risk of PND may improve maternal and child health outcomes and the detection of IPV might increase a woman and her children’s safety. However, the challenge is that often the ethical justification is based on a belief that positive benefit will result from participation, but these benefits cannot be guaranteed as an effect because this is determined at study completion.

Some have thus questioned the use of treatment as usual (TAU) as a control condition particularly in psychosocial RCTs where serious risks may be identified in a comparison group, for example pregnant black women at risk of serious, even life-threatening harm from IPV, because not everyone who screens positive is provided with an intervention, follow up or wait list control option [26]. On the other hand, to remain ethically committed to participants, TAU health care professionals may be provided with further information about patients to offset risk. Thus, the comparison is not really equivalent to ordinary health care and ecological validity is disrupted because components of TAU have been changed [25,29]. The exact nature of TAU is also difficult to document since health care providers for those in the comparison group could be quite different and frequency of contact could differ [32]. Moreover, for some conditions TAU may be seen as substandard care or similar to receiving no treatment which introduces a tension between the potential public benefits (justice) of establishing intervention effectiveness and the need to minimize risks for individuals (beneficence) [29]. Testing for psychosocial intervention effectiveness may therefore be difficult to ascertain within the RCT design that uses TAU. When screening is used with such vulnerable populations who are identified as at risk of depression, PND or IPV; it seems that there is little option but to provide a form of augmented TAU care to remain ethical.

There is no doubt that the common justification for incorporating screening to identify eligible study participants is related to beneficence or the hypothesized benefits (for individuals and the population) that could result from determining the efficacy of an intervention. However, one trial completed to evaluate the effectiveness of screening to recruit participants to a psychotherapy RCT determined the method to be ineffective [33]. Similar to studies of IPV screening, whilst the majority of women completed the screening questionnaire, only a minority affected agreed to be contacted and assessed. Of those who declined further contact the reasons included being too shy, not thinking that they were depressed enough, not wanting to be labeled and the GP telling them that they were not depressed [33]. Studies have found that non-participation is elevated in the presence of a history of mental disorders (or psychosocial issues as caused by IPV experiences) [34]. Therefore, determining some of the reasons why non-participation occurs is an important ethical consideration for considering the use of screening for IPV and depression in intervention studies.

### Screening, new diagnoses and disclosure: doing no harm?

Under the principle of non-maleficence we are obliged to avoid harm, however, there is a potential for harm to occur in these three studies directly related to completing a screen. This is not only because of the study designs where not everyone will receive a potentially beneficial intervention, but also because screening brings with it new information and possible burdens that did not exist before. This information may change a person’s sense of self, their identity and introduce new health care pathways not previously considered. Whilst the possible benefits of an intervention must be balanced against potential risks involved in each study, before participants even engage in an intervention, being screened to determine eligibility has introduced a new and immediate risk of harm. For community based studies in depression and PND this relates to the burden of new information about a diagnosis since screening can be prospective and participants do not necessarily have an existing diagnosis of depression [27]. Participants may then receive new health information that causes discomfort or new health care pathways not anticipated. New diagnoses introduce the potential for those same individuals to harm others by not disclosing diagnostic information about depression or PND to family members [35]. There is possible harm with detecting risk of PND because the changes to a person’s self understanding may compromise a mother’s resiliency and feelings of agency. For those who disclosed in IPV studies there is also the risk that if assigned to the intervention, it may be unsuccessful. Participants may be disappointed in their allocated status or in interventions, and further harmed by IPV disclosure as women might fear retaliation from partners or the involvement of police or other government agencies such as child protection.

In all three of our study examples screening has already occurred and alerted the participant to a potential issue that may require attention before they consent to participate in the larger intervention. This new information may influence people’s decision-making and contribute to increased expectations of receiving a new treatment or being provided with other supports which is not the case with a RCT design. Indeed, others have noted that there is a risk of increased hopelessness and despair for people entering studies because when they are depressed, for example, they may feel that in spite of taking action they are not getting better [32]. Or, even though participants are informed of being assigned to a control group they can become demoralized or blame themselves when they do not receive the intervention [32].

Currently, the major justification that harm has been avoided comes down to the fact that a participant has provided informed consent. This emphasis on autonomous decision-making obscures these risks of harm that we have outlined.

### Screening and autonomy: informed decision-making?

Respect for autonomy is viewed as the obligation to respect the decision-making capacity of the autonomous person which is represented in intervention studies through participants giving their informed consent. Krantz et al. [36] have argued though that informed consent is difficult to achieve with screening, since scientific validity is not a topic that is easy to understand and the public generally do not understand the problems associated with tool design and interpretations. Indeed, within primary care populations participants may often complete a screening instrument to determine eligibility, but their awareness of the tool being a screener for depression, PND or IPV can be limited.

There is a prevailing view that screening tools are not therapeutic (particularly when used to identify eligibility in an intervention study) and so they are neutral, value-free tools [10]. In the first instance, screening tools function as ‘technologies of trust,’ that is, from the recipients’ point of view the numerical value produced from a tool can be seen to offer an objective and legitimate view whereby results are accepted regardless of the potential for a false positive result [37]. This trust in the tool may well influence decisions that people subsequently make to participate in research, it may influence expectations and we need to be aware that this might contribute to the possible risk of demoralization, hopelessness or despair, as we outlined above.

In addition, it is not certain that people do make informed decisions when they are vulnerable (during pregnancy or elderly and possibly depressed). None of the three studies we have examined call into question the ability of participants to make their own decisions to complete screening questionnaires, but the settings where they are conducted increase their vulnerability. For example, women in their first pregnancy were targeted in Brugha et al’s intervention [24] and recruited from hospitals, those pregnant and disadvantaged with a high risk of IPV were targeted in El-Mohandes’ et al’s intervention [26]. All of the participants might have been said to be susceptible to ‘decisional pressures’, that is, when a person is in unfamiliar surrounds like a hospital they are ‘highly suggestible to figures in authority, even when told to do things that they would not ordinarily do’ [38]. Elderly patients and any patient who has attended a primary care practice for a long period of time also develop trust in the setting and their providers. This trust may influence how they decide and whether screening is an acceptable practice for them. Moreover, when eligibility is determined within clinical settings such as hospitals or primary care sites sometimes participants do not realize that the tests are not required for their medical care. When we focus on autonomy as the ability to simply provide informed consent we fail to appreciate how one’s environment can shape and affect decisions [39].

### Screening and intervention design: just benefits?

Under the principle of justice there is the obligation to fairly distribute the benefits and risks, Beauchamp and Childress also suggest that the principle of justice requires a commitment to ‘equals being treated equally and unequals unequally’ [1]. So is it fair distribution of benefits and risks to screen participants at risk of psychosocial conditions and only provide an intervention to one group? Some argue that one response to this is to offer treatments where effectiveness has been established to participants when a study is complete (wait list control) [32]. In his paper explaining the CABLES model to assess and minimize risk in research, Koocher notes that researchers need to be mindful that participants may have undergone ‘critical development periods’ which vitiate the possible benefits of an intervention [40]. Koocher proposes that six domains must be attended to and evaluated to fully explore potential risks for participants. These include: cognitive, affective, biological, legal, environmental and social (CABLES). As we have highlighted above, people who have been screened for IPV, PND or depression may now have knowledge that impacts on any one or more of these domains.

Currently randomization is ethically justified using the principle of equipoise ‘such that across the research community there should be no clear belief regarding the relative efficacy of one treatment condition over another’ [41,42]. However equipoise is premised on research design being an unbiased and objective endeavor, which is questionable and more suited to clinical drug trials or interventions testing the effectiveness of a pharmacological medication versus a complementary therapy for example. In the case of psychosocial research designs a great deal of effort is put into identifying all of the available evidence to support testing an intervention’s effectiveness -- which does raise some doubt about how much ‘treatment’ uncertainty may exist. Indeed Miller and Brody, along with others, have advocated for the abandonment of clinical equipoise and proposed that a clear distinction between therapeutic practice (clinical or medical care) and clinical research is necessary [43-46]. For unequals to be treated unequally in the three studies, they must also receive benefits of being identified as at-risk of depression, PND or IPV.

## The possible limits of principlism

Whilst large scale public health interventions may be assessed using a range of ethical theories, research intervention studies are most often subjected to ethical assessment by the application of the four principles. Critics of principlism argue that the principles foster a checklist approach to ethical problems [47], they emphasize individual autonomy to the exclusion of context and the inter-subjective nature of how people live their lives, and how this can affect decision-making [48], and they are universally applied [49]. Some feminists and others have critiqued the sense of impartial deliberation, and focus on rationality and reasoning espoused in principlism [50]. Criticisms have also been made about its reliance on deductive rather than inductive conclusions. Beauchamp and Childress counter criticisms about deductive versus inductive approach with the addition of ‘interpretation, specification, and balancing of the principles in order to formulate policies and decide about cases’ [51].

Even though we have attempted to specify and examine how the principles can be applied to the ethical issues of using screening within intervention studies, it is still unclear how we can resolve some of the ethical tensions that have been raised. First, in the current research context we cannot categorically rule out the use of TAU as a control condition for psychosocial interventions since effectiveness of an intervention must be established and this is the most scientifically rigorous method that currently exists. This brings into question whether benefits and risks can be distributed fairly when screening is used to identify people at risk of depression, PND or experiencing IPV if no follow up or wait list control is provided. In addition, we know that there may well be substantive benefits to result from some interventions if they are established, but whether these unknown benefits outweigh the immediate potential for harm for individuals that new health information can bring is yet to be determined. Since principlism leaves us at an impasse on the issue of using screening within intervention studies, we now deploy a narrative and relational approach to these issues in an attempt to resolve tensions and explore the issues in greater depth.

## Ethical issues raised by the three study examples – the view of narrative and relational ethics

Since the 1980s, narrative approaches have gained popularity and introduced the importance of personal stories and identity in medical and research ethics. Coupled with this has been greater interest in narrative within bioethics, although some have argued that narrative is sometimes positioned as a helpmate to philosophy and its importance as a field of inquiry in its own right is sidelined [52]. Lindemann has noted that narrative approaches ‘challenge the assumption that ethics has primarily to do with right conduct among strangers, is universalizable and favors no one’ [53]. Narrative emphasizes who people are, their relationships and how these are to be valued if they are important, this is different to the highly individualized focus of principlism and its potential to provide an abstracted, de-contextualized view of ethical issues. Lindemann adds that narrative approaches to ethics contest the view that moral principles are law like, universal and unyielding. Instead, they are ‘modifiable in light of the particulars of a given experience or situation…[and] these particulars either naturally take a narrative form or must be given a narrative structure if they are to have meaning’ [53].

Central to this sense of humans as narrative beings is a view of ethics as relationally constituted. Not only is narrative and identity intimately connected, but we exist in relation with others. Drawing on Young’s work, Kenny et al. [4] propose that ‘relational persons develop and deploy their values within the social worlds they inhabit, conditioned by the opportunities and obstacles that shape their lives according to the socially salient features of their embodied lives (e.g. their gender, race, class, age, disability status, ethnicity)’. These socially and relationally salient features are fundamentally important to understanding who we are as humans and they provide us with a different viewpoint from which to assess ethical issues like using screening within an intervention study.

Table 1 outlines some ethical considerations that have emerged following our analysis of this issue from the perspective of principlism. Some of the issues are about the need to explore the contextual story about screening – where does it occur? What are the important things to consider in the environment and setting where screening is being conducted? How does our socio-cultural world impact on this experience of being screened? Other matters relate to the need to acknowledge the role of identity in deciding to participate in a study - individual identity does matter and life experiences influence a person’s decision to participate. In addition to this, our relational world shapes how we may respond to the experience of being screened to determine eligibility for a study and raises new questions. From here, we consider some of these narrative and relational issues to help us better understand the ethical complexities of using screening within interventions studies for IPV, depression and PND.

**Table 1:** Ethical considerations brought to light considering a narrative and relational approach

Environment/ Setting Political and policy context Organizational support / professional training to conduct screening Provision of resources for follow up Access to care and services Decisional pressures Design of screening tools Design of studies	Socio-Cultural Context Labeling groups ‘at-risk’ Victim Blaming Different cultural understandings of depression and intimate partner violence Cultural appropriateness of screening tools
**Individual Issues** Dealing with new health information Personal values Disruption of identity Stigma and Shame of new diagnosis	**Relational Issues** Relational responsibilities Impact on families Do I tell people close to me Attitudes of health professionals

### Individual and relational Issues

Taylor argues that experience, interpretation and the significance this forges has the possibility of changing a person’s self-understanding, even if only partially [35]. People are not neutral spectators and so their experience of being screened and the meaning of a diagnosis or label that could result is likely to be evaluated in the context of their life [35]. Given this, it is important that we fully appreciate who participants in our studies are and consider how screening before an intervention may impact on their identities and relationships. To appreciate this means having available accounts of how participants experience events and issues outside of the interventions in question; currently findings from interventions report only on the majority view. A full ethical assessment will require an assessment of the various components of research studies which includes methods that are used to determine eligibility and not only a report of the outcomes achieved.

As Scheman notes, human research subjects are both the objects of narrative and narrators and it is ‘as narrators that research subjects (and other people and things related to those objects) will be normatively autonomous’ [48]. To fully incorporate this perspective into intervention research means that there needs to be an epistemic move in the direction of interrelationship and interdependency. This will require researchers and those to be researched to find ways to work collaboratively in relationships rather than as falsely separated beings because of the issues of blurred boundaries and ‘contamination of findings’. What is at risk currently is that we are treating the ‘congeries of symptoms that reveal themselves to diagnostic tools’ manifest as depression, PND or IPV and we are forgetting ‘who’ the people are who are engaged in studies [48].

From the development of narrative oriented accounts that explore people’s experiences of being screened it is possible to determine to whom participants feel morally obligated and how their existing relationships may be impacted upon by new information. Since knowing new information brings with it the question of responsibility about whether that information needs to be shared with family members or significant others in people’s lives, researchers would do well to understand the consequences of new knowledge for participants beyond their rights as individuals. In addition to this, women who leave situations where they are experiencing IPV are often highly dependent on supports offered by friends and family; thus intervention studies impact beyond the individual. This means coming to see decision-making as a relational act that is dependent not only on capacities and competencies but also fundamentally premised on our existence as dialogical (interdependent) beings.

The act of screening needs to also be explored from the relational context within which it is occurring. There have been reported benefits from women about having postnatal depression acknowledged by a health professional through screening but further consideration needs to be given to when this occurs. Delivering a screen during a hospital or midwifery consultation could be interpreted by women as an essential element of their medical care. We also have not explored how screening impacts on professionals and their views of its use within intervention studies. This is important to consider since a study examining US emergency nurses showed nurses as willing to screen patients who present with injury for IPV, but there continued to be a reluctance to implement the practice [54]. This reluctance may lie with the personal values or victimization experiences of nurses or other factors we are yet to determine such as the influence of the wider social narrative, ‘IPV is not my business because it is a private matter’ [54]. Considering the broader narratives that shape where screening takes place is important.

### Environment, setting and socio-cultural context

Although one study reported that no safety concerns resulted from IPV screening of patients, it is important to ask if the concept of ‘safety concern’ adequately captures the possible harm associated with screening for IPV? [7] It may be more appropriate to explore the issue of harm in a way that enables the subjective experiences of those being screened for both depression and IPV to come forward. For example Koziol-McLain et al. [54] found in their interviews with women screened for IPV that while most stated it was fine to be asked questions concerning IPV, women expressed feelings of surprise, shame and embarrassment when interviewed about their experience. One woman was quoted as saying that she nearly started crying when she was asked ‘those things’ but attributed feeling comfortable because of the person interviewing her; another said that although it is important to be asked they had not really dealt with ‘those feelings’ [54]. Bacchus et al [55] found breaches of confidentiality and other harms as a result of their screening intervention. This indicates that ‘safety concerns’ are only one part of the question about harm, beyond the individual is the relational context in which this asking occurs and the reality that the previous experience remains an integral part of the person’s identity (it is embodied).

Little attention has been given to the applicability of screening tools in socially and culturally diverse populations. While screening tools are increasingly being validated within specific ethnic populations [56], the possibility of false positives and false negatives from screening remains high. Moreover, there are questions as to how well tools capture the culturally nuanced ways in which depression and IPV experiences are expressed. It may also be that cultural background influences the preferred mode of how to ask about these sensitive issues and there may be culturally shaped attitudes and beliefs to screening and its results that need to be considered.

## Where to from here?

Table 2 lists some narrative and relationally driven questions that we have arrived at from this analysis; the questions are based on the important issues screening raises which are outlined in table 1. We use the English language interrogative pronouns – whom, who, whose, what and which to develop some questions that could be used to consider the issues. The pronouns are deployed intentionally because they are narrative based and they assist in drawing our attention to the four main areas that can be overlooked from a view of principlism. If we ask a question about what and which we can explore what the environmental and socio-cultural issues are and which issues require further consideration before screening is employed. If we ask some deeper questions about who our participants may be and to whom they may be obligated, we can identify whose interests may or may not be being represented in a study. Basing our questions on interrogative pronouns is premised on our day to day use of these words to ask questions about things that we are not yet aware of. These questions, we feel, are not the only solution to dealing with the ethical complexities of using screening within intervention studies. However, they provide a starting point for shifting the focus toward some of the deeper concerns that incorporating screening raises and the need to explore these in greater depth so that we can modify moral principles in light of the particulars of the situation as Lindemann advocates [53].

**Table 2:** Narrative and relationally driven questions to interrogate the use of screening in intervention studies

1. *To whom am I morally obligated? Who are the individuals and who matters to them?* Who is screening intended for? Is the group in question vulnerable? Do we understand who the people are who might participate in the intervention? Do we understand what matters to them and how this will shape their perceived obligations? Will the individuals be likely to feel stigmatized? Will there be worry and concern about whether to tell others about a potential diagnosis or their situation? 2. *Whose interests are being represented?* Whose voice is prominent in the research findings? Do we hear about those who may not wish to participate as much as those who do? What happens to the people who are identified as at-risk but do not receive further supports, follow up or interventions? What happens to participants when an intervention is not effective? 3. *What environmental and socio-cultural factors need to be accounted for?* Who is involved in delivery and receipt of screening tools? What cultural values are expressed about the practice of screening and the conditions being screened for? What is the nature of the relationships of those involved in screening? What are the environmental features that are likely influence decision-making? Is the tool culturally appropriate? What social factors are important to consider? What cultural understandings about conditions being screened for are important to know about in advance? 4. *Which study design and which tools do we use?* Which study designs are ethically justifiable with vulnerable populations? Which screening tools do we select from and how do we account for their shortfalls? Which processes can be incorporated to ensure that we focus on who our participants are instead of the symptoms revealed by diagnostic and identification tools?

## Summary

The ethical complexities of using screening to identify eligible research participants and recruit people to intervention studies for IPV, PND and depression need further deliberation and debate by researchers, practitioners, research participants and the broader public. Before screening is incorporated as a method to identify eligible participants or to recruit, it may be beneficial to ask if we truly understand who our participants are and whether their conditions and vulnerability affect the type of study designs we ought to use. Based on our assessment, since screening with vulnerable groups increases vulnerability through the identification of risk, there seems little to no choice but to offer an augmented form of treatment as usual or a wait list control.

We have proposed a set of simple, interrogative questions that are narrative and relationally driven. These questions will assist to further evaluate the potential impacts of using screening to identify eligible research participants and can supplement factors that are considered by applying a principle based approach. We have argued that the justification most commonly identified from an assessment of screening using principlism is based on the potential benefits of the intervention. This needs to be re-evaluated in light of the possible harms that individuals could experience from knowing they are at risk of depression or postnatal depression or making significant life changes because of being invited to participate in an IPV intervention. Rather than leave the ethical evaluation to the question of whether a person’s autonomy was respected, future intervention studies might do well to consider the matter of using screening by asking some of the questions we have put forward. This requires seeing people as situated within their social and cultural contexts and existing within relationships that are likely to be affected by the results of screening information. New information may burden people and engagement in studies change individuals and those with whom they exist in relation, even if only partially.

## Competing interests

The authors have no competing interests to declare.

## Authors’ contributions

VP, JY and AT conceived the ideas for this paper. VP, JY and AT carried out the ethical analysis of screening for depression and IPV and its use within intervention research studies from the perspective of principlism. VP developed the analysis of the issues using alternative ethical theories. All authors have read and approved the final manuscript.

## Supplementary Material

Additional file 1 **Table:** Three examples of intervention studies using screening as a component. Click here for file

Additional file 2 **Table:** Screening for depression and IPV – the guidance offered by principlism. Click here for file

## References

[B1] Beauchamp TL Childress JF Principles of biomedical ethics 2009 6 New York: Oxford University Press

[B2] Beauchamp TL Methods and principles in biomedical ethics J Med Ethics 2003 29 269 274 10.1136/jme.29.5.269 14519835 PMC1733784

[B3] Bloch S Green SA An ethical framework for psychiatry Br J Psychiatry 2006 188 7 12 10.1192/bjp.188.1.7 16388063

[B4] Kenny NP Sherwin SB Baylis FE Re-visioning public health ethics: a relational perspective Can J Public Health 2010 101 9 11 20364529 10.1007/BF03405552 PMC6974005

[B5] Childress JF Faden RR Gaare RD Gostin LO Kahn J Bonnie RJ Kass NE Mastroianni AC Moreno JD Nieburg P Freeman MDA. Burlington, VT Public health ethics: mapping the terrain The Ethics of Public Health 2010 1 Ashgate Pub. Co. 55 64 10.1111/j.1748-720x.2002.tb00384.x 12066595

[B6] DeVries R Kane Low L Bogdan-Lovis E Lindemann H, Verkerk M, Walker MU Choosing surgical birth: desire and the nature of bioethical advice Naturalized bioethics: toward responsible knowing and practice 2010 Cambridge; New York: Cambridge University Press 42 64

[B7] MacMillan HL Wathen CN Jamieson E Boyle MH Shannon HS Ford-Gilboe M Worster A Lent B Coben JH Campbell JC McNutt LA Screening for intimate partner violence in health care settings: a randomized trial J Am Med Assoc 2009 302 493 501 10.1001/jama.2009.1089 19654384

[B8] Shakespeare J Blake F Garcia J A qualitative study of the acceptability of routine screening of postnatal women using the Edinburgh Postnatal Depression Scale The British journal of general practice : the journal of the Royal College of General Practitioners 2003 53 614 619 PMC1314675 14601337

[B9] Buist A Condon J Brooks J Speelman C Milgrom J Hayes B Ellwood D Barnett B Kowalenko N Matthey S Acceptability of routine screening for perinatal depression J Affect Disord 2006 93 233 237 10.1016/j.jad.2006.02.019 16647761

[B10] Spangaro J Zwi AB Poulos R The elusive search for definitive evidence on routine screening for intimate partner violence Trauma, violence & abuse 2009 10 55 68 10.1177/1524838008327261 19056688

[B11] Pencheon D Guest C Melzer D Gray M Oxford handbook of public health practice 2001 Oxford: Oxford University Press

[B12] Taft A To screen or not to screen - is this the right question?: quality care, intervention and women's agency in health care responses to partner violence and abuse Women Against Violence: An Australian Feminist Journal 2001 41 46

[B13] Flitcraft A From public health to personal health: violence against women across the life span Ann Intern Med 1995 123 800 802 7574199 10.7326/0003-4819-123-10-199511150-00009

[B14] Hegarty K Gunn J Chondros P Small R Association between depression and abuse by partners of women attending general practice: descriptive, cross sectional survey BMJ 2004 328 621 624 10.1136/bmj.328.7440.621 15016694 PMC381136

[B15] Vik K Aass IM Willumsen AB Hafting M "It's about focusing on the mother's mental health'': screening for postnatal depression seen from the health visitors' perspective--a qualitative study Scand J Public Health 2009 37 239 245 10.1177/1403494808100275 19164429

[B16] Gilbody S Sheldon T House A Screening and case-finding instruments for depression: a meta-analysis Can Med Assoc J 2008 178 997 1003 10.1503/cmaj.070281 PMC2276549 18390942

[B17] Hickie IB Davenport TA Ricci CS Screening for depression in general practice and related medical settings Med J Aust 2002 177 Suppl S111 116 12358569 10.5694/j.1326-5377.2002.tb04869.x

[B18] Paulden M Palmer S Hewitt C Gilbody S Screening for postnatal depression in primary care: cost effectiveness analysis BMJ 2009 339 b5203 10.1136/bmj.b5203 20028779 PMC2797050

[B19] Pignone MP Gaynes BN Rushton JL Burchell CM Orleans CT Mulrow CD Lohr KN Screening for depression in adults: a summary of the evidence for the U.S. preventive services task force Ann Intern Med 2002 136 765 776 12020146 10.7326/0003-4819-136-10-200205210-00013

[B20] USPSTF Screening for family and intimate partner violence: recommendation statement Ann Fam Med 2004 2 156 160 15083857 10.1370/afm.128 PMC1466657

[B21] Feder G Ramsay J Dunne D Rose M Arsene C Norman R Kuntze S Spencer A Bacchus L Hague G How far does screening women for domestic (partner) violence in different health-care settings meet criteria for a screening programme? Systematic reviews of nine UK National Screening Committee criteria Health Technol Assess 2009 13 1 347 19272272 10.3310/hta13160

[B22] Moracco KE Cole TB Preventing intimate partner violence: screening is not enough J Am Med Assocn 2009 302 568 570 10.1001/jama.2009.1135 19654392

[B23] Taft AJ Hegarty KL Intimate partner violence against women: what outcomes are meaningful? J Am Med Assoc 2010 304 577 579 10.1001/jama.2010.1093 20682943

[B24] Brugha TS Wheatley S Taub NA Culverwell A Friedman T Kirwan P Jones DR Shapiro DA Pragmatic randomized trial of antenatal intervention to prevent post-natal depression by reducing psychosocial risk factors Psychol Med 2000 30 1273 1281 10.1017/S0033291799002937 11097068

[B25] Reynolds CF 3rd Degenholtz H Parker LS Schulberg HC Mulsant BH Post E Rollman B Treatment as usual (TAU) control practices in the PROSPECT Study: managing the interaction and tension between research design and ethics Int J Geriatr Psychiatry 2001 16 602 608 10.1002/gps.466 11424169

[B26] El-Mohandes AA Kiely M Joseph JG Subramanian S Johnson AA Blake SM Gantz MG El-Khorazaty MN An intervention to improve postpartum outcomes in African-American mothers: a randomized controlled trial Obstet Gynecol 2008 112 611 620 10.1097/AOG.0b013e3181834b10 18757660 PMC2935657

[B27] Degenholtz HB Parker LS Reynolds CF Trial design and informed consent for a clinic-based study with a treatment as usual control arm Ethics & behavior 2002 12 43 62 10.1207/S15327019EB1201_3 12171082

[B28] Alvidrez J Arean PA Psychosocial treatment research with ethnic minority populations: ethical considerations in conducting clinical trials Ethics & Behavior 2002 12 103 116 10.1207/S15327019EB1201_7 12171080

[B29] Arean PA Alvidrez J Ethical considerations in psychotherapy effectiveness research: choosing the comparison group Ethics & Behavior 2002 12 63 73 10.1207/S15327019EB1201_4 12171083

[B30] Saks ER Jeste DV Granholm E Palmer BW Schneiderman L Ethical issues in psychosocial interventions research involving controls Ethics & Behavior 2002 12 87 101 10.1207/S15327019EB1201_6 12171085

[B31] Campbell AV Charlesworth M Gillett G Jones DG Medical ethics 1997 2 Oxford ; New York: Oxford University Press

[B32] Street LL Luoma JB Control groups in psychosocial intervention research: ethical and methodological issues Ethics & Behavior 2002 12 1 30 10.1207/S15327019EB1201_1 12171079

[B33] Carter FA Carter JD Luty SE Wilson DA Frampton CM Joyce PR Screening and treatment for depression during pregnancy: a cautionary note Aust N Z J Psychiatry 2005 39 255 261 10.1080/j.1440-1614.2005.01562.x 15777362

[B34] Kessler RC Little RJ Groves RM Advances in strategies for minimizing and adjusting for survey nonresponse Epidemiol Rev 1995 17 192 204 8521937 10.1093/oxfordjournals.epirev.a036176

[B35] Kearns AJ O'Mathuna DP Scott PA Diagnostic self-testing: autonomous choices and relational responsibilities Bioethics 2010 24 199 207 10.1111/j.1467-8519.2008.00711.x 19222443

[B36] Krantz I Eriksson B Lundquist-Persson C Ahlberg BM Nilstun T Screening for postpartum depression with the Edinburgh Postnatal Depression Scale (EPDS): an ethical analysis Scand J Public Health 2008 36 211 216 10.1177/1403494807085392 18519287

[B37] Jansen YJ de Bont AA The role of screenings methods and risk profile assessments in prevention and health promotion programmes: an ethnographic analysis Health Care Anal 2010 18 389 401 10.1007/s10728-009-0141-0 20063197 PMC2970818

[B38] Lindemann H Lindemann H, Verkerk M, Walker MU Holding on to Edmund: the relational work of identity Naturalized bioethics: toward responsible knowing and practice 2009 Cambridge; New York: Cambridge University Press 65 79

[B39] Walker MU Lindemann H, Verkerk M, Walker MU Introduction: groningen naturalism in bioethics Naturalized bioethics: toward responsible knowing and practice 2009 Cambridge ; New York: Cambridge University Press 1 20

[B40] Koocher GP Using the CABLES model to assess and minimize risk in research: control group hazards Ethics & behavior 2002 12 75 86 10.1207/S15327019EB1201_5 12171084

[B41] Grant JB Mackinnon AJ Christensen H Walker J Participants' perceptions of motivation, randomisation and withdrawal in a randomised controlled trial of interventions for prevention of depression J Med Ethics 2009 35 768 773 10.1136/jme.2008.028035 19948934

[B42] Schlichting DE Destabilizing the 'equipoise' framework in clinical trials: prioritizing non-exploitation as an ethical framework in clinical research Nurs Philos 2010 11 271 279 10.1111/j.1466-769X.2010.00455.x 20840138

[B43] Miller FG Brody H Clinical equipoise and the incoherence of research ethics J Med Philos 2007 32 151 165 10.1080/03605310701255750 17454420

[B44] Veatch RM The irrelevance of equipoise J Med Philos 2007 32 167 183 10.1080/03605310701255776 17454421

[B45] Gifford F So-called "clinical equipoise" and the argument from design J Med Philos 2007 32 135 150 10.1080/03605310701255743 17454419

[B46] Miller FG Brody H A critique of clinical equipoise. Therapeutic misconception in the ethics of clinical trials Hastings Cent Rep 2003 33 19 28 12854452

[B47] Harris J In praise of unprincipled ethics J Med Ethics 2003 29 303 306 10.1136/jme.29.5.303 14519841 PMC1733790

[B48] Scheman N Lindemann H, Verkerk M, Walker MU Narrative, complexity, and context: autonomy as an epistemic value Naturalized bioethics: toward responsible knowing and practice 2009 Cambridge; New York: Cambridge University Press 106 124

[B49] Lindemann H Verkerk M Walker MU Naturalized bioethics: toward responsible knowing and practice 2009 Cambridge; New York: Cambridge University Press

[B50] Edwards SD Three versions of an ethics of care Nurs Philos 2009 10 231 240 10.1111/j.1466-769X.2009.00415.x 19743967

[B51] Beauchamp TL Principlism and its alleged competitors Kennedy Inst Ethics J 1995 5 181 198 11645305 10.1353/ken.0.0111

[B52] Chambers T The fiction of bioethics: a precis Am J Bioeth 2001 1 40 43 11808596 10.1162/152651601750079050

[B53] Lindemann Nelson H Lindemann H Introduction: how to do things with stories Stories and their limits: narrative approaches to bioethics 1997 1 New York: Routledge

[B54] Koziol-McLain J Giddings L Rameka M Fyfe E Intimate partner violence screening and brief intervention: experiences of women in two New Zealand health care settings J Midwifery Womens Health 2008 53 504 510 10.1016/j.jmwh.2008.06.002 18984506

[B55] Bacchus LJ Bewley S Vitolas CT Aston G Jordan P Murray SF Evaluation of a domestic violence intervention in the maternity and sexual health services of a UK hospital Reprod Health Matters 2010 18 147 157 10.1016/S0968-8080(10)36526-8 21111359

[B56] Depression: The NICE guideline on the treatment and management of depression in adults (updated edition). National Clinical Practice Guideline 90 http://www.nice.org.uk/nicemedia/live/12329/45896/45896.pdf

