# Supplementary material for: Ethical complexities of screening for depression and intimate partner violence (IPV) in intervention studies
Source: BMC Public Health. 2011 Nov 25;11(Suppl 5):S3. doi: 10.1186/1471-2458-11-S5-S3 (PMC3247026; doi:10.1186/1471-2458-11-S5-S3)
Supplement: Additional file 1 — Table: Three examples of intervention studies using screening as a component. [file 1471-2458-11-S5-S3-S1.doc]

# S3 Additional file 1

Three examples of intervention studies using screening as a component

| **Study** | **Design** | **Screening component** |
| --- | --- | --- |
| Pragmatic randomized trial of antenatal intervention to prevent post-natal depression by reducing psychosocial risk factors (Brugha et al).  *Participants:*  Primiparous women identified in pregnancy at increased risk of postnatal depression. | *Design:* Randomized controlled trial.  *Control:*    *Intervention:* ‘Preparing for Parenthood’ package based on models of social support and problem solving; multifaceted strategies. | *Screening to recruit:*  All mothers in their first pregnancy during 12 to 20 week gestation and meeting eligibility criteria (16 y/o, reside close to hospital, understood English) asked to complete a screening questionnaire to identify possible antenatal depression. Following consent, those women scoring as depressed were randomized into intervention and control groups. |
| An intervention to improve postpartum outcomes in African American Mothers: A randomized controlled trial (El Mohandes et al)  *Participants*:  Pregnant African American and Latina women who were Washington residents aged 18 or more, and who were 28 weeks gestation or less. | *Design*: randomized controlled trial  *Control:*  *Intervention*: a multi-modal counseling and education intervention (using Cognitive Behavioral Therapy and Stages of Change) delivered in minimum 4 and max 8 sessions and two optional postpartum to reduce damaging problems, including IPV, depression, smoking and smoking exposures and to evaluate the intervention’s impact on any reduction of pre-term or low birth weight pregnancy outcomes and lower morbidity and mortality. | *Screening to determine eligibility for intervention study:*  Eligibility was assessed by women completing an Audio Computer-Assisted Self Interview (A-CASI) screening survey to identify SES and a range of psychosocial behavioral risk factors including depression, smoking, and IPV. This required initial consent. Once assessed as eligible, RAs then provided more information at next visit to service and a second written consent to participate and complete the baseline interview. Women were then randomized into intervention and control groups. |
| Treatment as usual (TAU) control practices in the PROSPECT Study: managing the interaction and tension between research design and ethics (Reynolds et al).  *Participants:* representative sample of older patients in primary care. | *Design:* Randomized controlled trial.  *Control:* Treatment as usual  *Intervention:* assessing effectiveness of an intervention in preventing and reducing suicide ideation and behavior, hopelessness and depressive symptoms by including mental health specialists at practices. | *Screening prospectively to determine eligibility*:  Patients screened by telephone using the Centre for Epidemiological Studies of Depression (CES-D). Patients who screened positive (≥11 on the CES-D) invited to meet research assistant on their next visit to the practice and learn more about the study and if willing undergo formal assessment for eligibility. |
