# Supplementary material for: Ethical complexities of screening for depression and intimate partner violence (IPV) in intervention studies
Source: BMC Public Health. 2011 Nov 25;11(Suppl 5):S3. doi: 10.1186/1471-2458-11-S5-S3 (PMC3247026; doi:10.1186/1471-2458-11-S5-S3)
Supplement: Additional file 2 — Table: Screening for depression and IPV – the guidance offered by principlism. [file 1471-2458-11-S5-S3-S2.doc]

# S3 Additional file 2

Screening for depression and IPV – the guidance offered by principlism.

|  | **Beneficence**  ***The obligation to provide benefits and to balance benefits against risks*** | **Non-maleficence**  ***The obligation to avoid causing harm*** | **Autonomy**  ***The obligation to respect the decision-making capacity of the autonomous person*** | **Justice**  ***The obligation of fairness in the distribution of benefits and risks*** |
| --- | --- | --- | --- | --- |
| **Depression**  **Studies** | Depression confers serious risks of harm for particularly mothers, children and society. Incorporating screening in a study will identify those at-risk so that they potentially receive appropriate treatment and management.  Screening for those at-risk to identify potential depression and test an intervention provides a benefit because an effective intervention may be determined that prevents future risk of individual and social suffering and improves maternal and child outcomes. | Not screening is potentially more harmful than associated stigma from receiving a new diagnosis: as sufferers live their lives without identification of their problem and this potentially confers greater distress/disability/illness with greater ‘cost’ to people and the community.  Screening is simply a tool that measures a problem and so causes minimal harm when administered. Using a tool to determine eligibility for recruitment therefore also causes minimal harm when administered. | Although patients rarely make truly informed decisions about being screened, they can determine whether they accept treatment or not by agreeing or not to participate in an intervention study.  Autonomy is thus respected in intervention studies because participants provide their informed consent. | All patients have an equal chance of being screened and will be no worse off than if screening had not been conducted. Future populations will benefit by the reduced burden of depression on the health and social system if interventions are determined to be effective.  More will be learned about treatment effectiveness to distribute the benefits fairly. |
| **IPV**  **Study** | IPV confers serious risks of harm but the risk of harm from IPV is higher than the risk of harm from being screened. Screening for IPV allows victims potentially to receive support and prevent serious injury in the future.  Identification balances the risks of IPV disclosure against the benefits of prevention and reduction in physical psychological harm. | Screening to determine eligibility may do harm to a few individuals. There are risks of disclosure of IPV for intervention and comparison participants. Further possible harm could result with the potential for stigma with disclosure.  Screening avoids the potential for harm caused by not treating an existing and profound problem to individuals and society. | Victims can judge whether to disclose or not and whether to take further action.  Autonomy is respected in intervention studies because participants provide their informed consent. | All patients have an equal chance of being screened and will be no worse off than if study had not been conducted.  Effective interventions could reduce the burden for victims, their families and communities, but not everyone receives the intervention. |
